# Development of a comprehensive sustainability index for extractive and mining companies: Integrating the Sustainable Development Goals

**DOI:** 10.1016/j.heliyon.2025.e41975

**Published:** 2025-01-15

**Authors:** Marta Fernández-Hernández, Pedro Mora, Marcelo F. Ortega, Juan Pous Cabello

**Affiliations:** a Departamento de Ingeniería Geológica y Minera, Escuela Técnica Superior de Ingenieros de Minas y Energía, Universidad Politécnica de Madrid, C/ Alenza 4, 28003, Madrid, Spain; b Laboratorio de Ingeniería de Proyectos, Escuela Técnica Superior de Ingenieros de Minas y Energía, Universidad Politécnica de Madrid, C/ Alenza 4, 28003, Madrid, Spain; c Escuela Técnica Superior de Ingenieros de Minas y Energía, Universidad Politécnica de Madrid, C/ Alenza 4, 28003, Madrid, Spain

**Keywords:** Sustainability, Extractive industry, Sustainability index, Sustainable development goals

## Abstract

Sustainability in the extractive and mining industry has become a key global concern, necessitating alignment with the United Nations Sustainable Development Goals (SDGs). This research introduces an innovative, scientific index-based assessment methodology designed to quantify the contribution of companies in these sectors to the SDGs. The methodology leverages a metric index scoring system to evaluate and enhance their sustainability performance.

The methodology involves scoring a series of parameters associated with each SDG, resulting in individual indices that are rescaled for comparability. This comprehensive approach also integrates environmental, social, and economic factors. The practical application of this index is critical, providing a step-by-step guide for extractive and mining companies to adopt and utilize the sustainability index, aiding in compliance with future regulations.

The results of this study show that the system of SDGs, targets, aspects, and metrics is naturally weighted towards social issues. It has also been observed that the SDG system, with respect to the targets, is not balanced. This same heterogeneous behavior occurs for aspects. This implies that the system is naturally weighted, which directly affects the comparison of contribution. The proposed methodology provides a process of normalization of indices that avoids this natural weighting, allowing the comparison of results.

This study advances a comprehensive methodology for evaluating extractive companies' contributions to the SDGs, incorporating multiple dimensions and expert opinions, and its adaptability allows for continuous improvement by incorporating new parameters. The Susceptibility Index helps management assess contributions on a scale of 0–10, facilitating ongoing improvements and anticipating regulatory changes.

This research enhances the assessment of extractive companies' SDG performance, promoting responsible practices. The open-access nature of the methodology enables any organization to conduct a sustainability analysis, fostering transparency and collaboration.

## Highlights

• The approach covers environmental, social, and economic factors for a holistic view of sustainability performance.
• No uniform method exists to assess how extractive companies contribute to sustainability and the SDGs.
• A novel methodology evaluates sustainability in mining, integrating the SDGs into the extractive industry's practices.
• By aligning with the SDGs, the method motivates companies to foster a sustainable, equitable, and fairer future.
• The index supports future sustainability reporting, benchmarking, and compliance with anticipated standards in mining.

## 1 Introduction

Throughout history, extractive endeavors have played a vital role in fostering societal progress, catering to various activities and necessities by extracting and refining mineral resources [1]. As civilizations evolved, the demand for products derived from these resources intensified, parallel to the advancement of societies. The twenty-first century has seen an exponential increase in resource exploitation, mirroring the economic growth of nations [2]. Moreover, modern factors such as the exponential growth of the world's population in the last 50 years, the fifth industrial revolution, renewable technologies or the Net Zero target, have driven the consumption of mineral resources to unprecedented levels. Specifically, during the last century the global use of materials increased 8-fold [3].

The extractive industry possesses the potential to exert both positive and negative influences on human development [4,5]. On the positive side, this sector can contribute to economic expansion, employment generation and infrastructure development, as well as serve as a basis for the development of innovative technologies for a more sustainable world. These aspects play a crucial role in improving well-being, living standards and the protection of our planet. By providing employment opportunities, generating public revenues through taxes and royalties, and encouraging economic activity in neighboring regions, it can contribute significantly to societal progress [6].

The extractive industry can also pose challenges to human development [7], particularly in developing countries or regions with weak governance systems. This is mainly because the policies developed in such regimes prevent the much-needed collaboration and participation of interested parties, leaving business decisions solely at the discretion of the governing bodies.

In contemporary society, extractive industries hold a prominent position, serving as a cornerstone across various sectors. Minerals are indispensable resources, catering to the needs of nearly every industry. Their value extends beyond their inherent material worth, as the natural environments from which they are extracted harbor immense potential.

The concept of sustainability is increasingly present in different aspects of our lives [8,9]. Sustainability and mining are two topics that are often discussed together due to the significant environmental and social impacts associated with mining activities ([7,10]). The classical theory of sustainability is based on three fundamental pillars (social, economy and environmental), known as the “three pillars of sustainability” [11]. The Brundtland Commission, in its report “Our Common Future” [12], played an important role in highlighting the notion of sustainable development and the interconnection between these aspects of sustainability. The extraction of natural resources is undeniably crucial for fostering economic growth so, it is imperative that such activities are approached with utmost caution, considering the potential environmental, social, and economic repercussions [13]. Upholding sustainability principles demands the responsible and efficient utilization of resources, preservation of ecosystems, fair distribution of benefits, and meaningful involvement of local communities in decision-making processes. Achieving these objectives requires a collaborative effort and dedication on the part of all interested parties, both those who experience the impacts, and the ones that contribute to them. The concept of sustainable mining revolves around the notion of conducting mining operations in a manner that mitigates adverse environmental and social effects while maximizing long-term advantages [14]. This approach entails the incorporation of principles of environmental stewardship, social responsibility, and economic viability into mining practices. By doing so, sustainable mining strives to strike a harmonious balance between resource extraction, environmental preservation and the well-being of local communities and future generations.

The creation of the ‘2030 Agenda for Sustainable Development’ and its Sustainable Development Goals (SDGs) were the result of a cooperative effort among various stakeholders [15]. In September 2015, the United Nations (UN) General Assembly adopted the SDGs, securing an unprecedented global commitment from 193 countries to eradicate poverty, protect the environment, and promote equality, peace, and justice [16]. The 17 SDGs address a wide range of environmental, social, and economic challenges, encompassing a total of 169 targets [17]. Since the establishment of the Mining and Metals for Sustainable Development (MMSD) initiative in the late 1990s [18], the mining industry has experienced a proliferation of sustainability endeavors. The SDGs provide a valuable framework for evaluating the corporate-level impacts of mining companies and their contributions to broader aspects of sustainable development [19]. Furthermore, the SDGs offer an opportunity for mining companies to build trust with host communities and make progress towards obtaining their ‘social license to operate’ [20].

With the increasing global demand for minerals and metals [21], the mining industry possesses significant potential to contribute to the SDGs. It can do so by providing essential raw materials for technological advancements, fostering economic growth and human development [22,23], generating royalties and taxes to support national government initiatives, creating employment opportunities, facilitating infrastructure development, and engaging in corporate social investments. Mining operations, including sites and associated processing plants, exert significant influences on local communities, economies, and ecosystems, often spanning long periods of operation, with both positive and negative ramifications. Achieving the SDGs necessitates collective and collaborative efforts from public and private entities [24], and mining companies are well-positioned to contribute, given their experience in working with governments, civil society, and development agencies to secure their license to operate [25]. Moreover, aligning a mining company with the SDGs not only enhances its corporate image but also opens doors to new markets, attracts investors, drives innovation, and fosters strategic collaborations. Companies like Rio Tinto have improved their reputation through transparency and social responsibility, reducing their environmental impact (SDGs 12 and 13), and contributing to the well-being of local communities (SDGs 1, 3, and 4). This not only builds trust among stakeholders but also facilitates access to capital interested in Environmental, Social, and Governance (ESG) criteria, ensures compliance with international regulations, and increases consumer preference for sustainable products. Additionally, innovation in sustainable technologies and processes (SDG 9) and the development of responsible new products have enabled Rio Tinto to improve efficiency and productivity. By strategically collaborating with governments and NGOs (SDG 17) and obtaining recognized certifications, Rio Tinto has enhanced its credibility and access to international markets [[26], [27], [28]]. As mining companies more frequently align their sustainability reporting with the SDGs, there is often a tendency to emphasize positive contributions while neglecting negative impacts that could obstruct progress toward the goals.

According to Ref. [29] inappropriate mining practices can result in deforestation, water and air pollution, and loss of biodiversity. More recently, studies such as [30] have shown that global mining expansion is leading to ecosystem degradation and habitat fragmentation, increasing the vulnerability of species to extinction. Effective governance and regulation are essential to mitigate these negative effects and promote sustainability.

Campbell [31] stresses the importance of transparency and accountability in the management of mineral resources. Initiatives such as the Extractive Industries Transparency Initiative (EITI) seek to improve governance in the sector by promoting disclosure of payments and benefits derived from natural resource extraction.

Extractive industry can be a significant source of income and economic development for developing countries. Sachs and Warner [32] argue that, if properly managed, natural resources can drive economic growth and improve social welfare. The literature also highlights the risks of the “resource curse,” where over-reliance on mining can lead to economic volatility, corruption, and social conflict. More recent research by Arezki and Gylfason [33] reaffirms these risks, noting that poor governance and corruption can undermine the potential benefits of natural resource wealth.

Monteiro et al. [34] discuss how mining companies can align their operations with the SDGs implementing sustainable practices, which are crucial in this process. The recent adoption of sustainability assessment frameworks, such as ESG, by mining companies has been vital to evaluate and improve their alignment with the SDGs. These assessments identify areas for enhancement and ensure that mining practices are consistent with the Sustainable Development Goals. Life cycle assessment (LCA) tools have been integrated to measure and reduce the environmental impacts of mining operations from a more holistic perspective. The Dow Jones Corporate Sustainability Index (DJSI) is one of the most globally recognized indices and measures the performance of companies in terms of sustainability. The Global Reporting Initiative (GRI) provides a framework for sustainability reporting that mining companies can use to communicate their ESG performance. Use of the GRI standards enables mining companies to transparently and consistently report their impacts and contributions to sustainable development. The Responsible Mining Index (RMI) assesses the sustainability policies and practices of mining companies in several key areas, including environmental management, social impact, corporate ethics and governance. The Yale University Environmental Sustainability Index (EPI) ranks countries according to their environmental policy performance. Although not specifically targeted at the mining industry, it provides useful context on environmental sustainability in the countries where mining companies operate. To illustrate how large-scale mining companies can contribute to the SDGs, ‘Mapping Mining to the Sustainable Development Goals: An Atlas' [35] showcased potential avenues for collaboration. Mancini and Sala also mapped the social impacts of mining to SDG targets, Global Reporting Initiative (GRI) indicators, and European Union (EU) policy [36]. Companies can use this information to improve their environmental management strategies and contribute to national sustainability goals.

Several research and scientific papers have developed sustainability indexes for the mining and extractive industries, including those by Refs. [37,38]. Additionally, there are studies that focus on specific sustainability issues within the mining sector, such as those conducted by Refs. [39,40] or [41]. Recently, work has been carried out in which sustainability indices have been developed, but only evaluating concrete targets for a specific industry sector [42], or even only considering the SDGs, ignoring the targets [43]. There are also initiatives in which the SDGs are grouped together, which does not allow the specific contribution of each SDG to be examined [44], or which consider all the SDGs, but use a weighting factor that adds subjectivity to the evaluation system [45]. However, there is no global methodology encompassing the SDGs and their targets applicable to the extractive and mining sector. This gap between the SDGs and their relationship to mining poses a challenge to the effective implementation of the SDGs in the mining industry ([25,46]). Because of this, the need arises to develop a working methodology that allows linking the UN SDGs with the extractive industry, offering the possibility of assessing an organization's contribution levels.

This work proposes an iterative evaluation methodology based on indexes that allows to understand, analyze and evaluate the contribution of an organization in terms of sustainability, and in co-creation with the United Nations SDGs. The aim of this work is not to penalize companies whose contribution is lower, but to encourage and motivate them to enhance and join efforts to address global challenges and foster a more sustainable and equitable world, as the United Nations wants to promote in its initiative “Transforming our world: the 2030 Agenda for Sustainable Development”.

After this Introduction section, this paper will present the materials and methodology proposed for obtaining a sustainability index for the extractive and mining industry. The results will also be analyzed and discussed to finally establish some conclusions and future lines.

## 2 Materials and methods

The evaluation of the performance of mining companies in terms of sustainability and corporate responsibility has become increasingly important in the last decade. In this context, various indices have emerged as fundamental tools for measuring and communicating companies' commitment to sustainability in the mining sector. These indices, some of which have been presented before, such as the SASB, DJSI, CDP, and RMI, offer different approaches and measurement criteria. The diversity in indicators, weightings, and data used in these indices, makes the results obtained not comparable with each other.

**Fig. 1:**
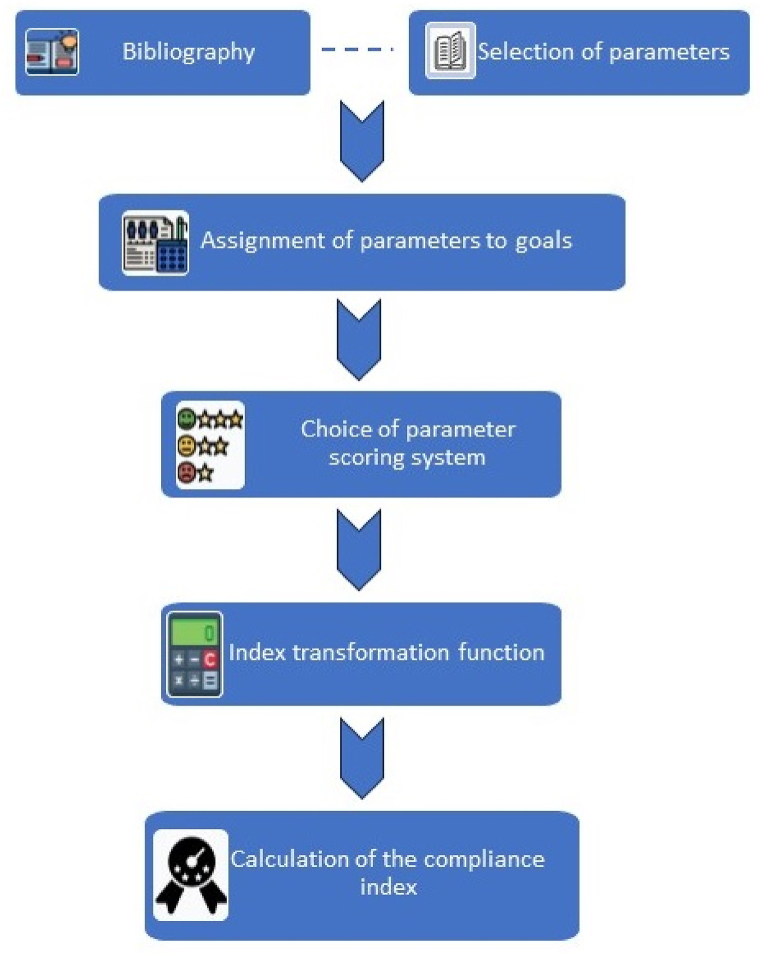
Work diagram with each of the main phases that have been carried out.

The main objective of this research is to develop a scientific index-based assessment methodology to quantify the contribution to the SDGs by an organization in the mining and extractive sector based on the quantification of a set of metric parameters. The developed methodology is based on the 17 Sustainable Development Goals (SDGs) and their 169 targets, using a set of parameters that will be scored. These parameters will be associated with each of the SDGs, resulting in an index for each of them, which will be normalized to make the results comparable with each other. Fig. 1 shows a diagram summarizing the main steps of this methodology.

The first step consists of reading and reviewing different indexes and reporting systems that can be used to evaluate sustainability in an extractive company. Specifically, the information provided by Mapping Mining to the SDGs: An Atlas [35] is reviewed, as well as the issues of the Responsible Mining Index [47] and the Dow Jones Sustainability Index [48]. The review of these reports and indexes has served to build a database of metric parameters that allow the evaluation of different aspects within the sustainability umbrella. In addition to this review of existing indices and reports, each of the SDGs has also been linked to the classic pillars of sustainability (economic, social and environmental) in accordance with the work done by Ref. [49]. This assignment also facilitates the link of parameters coming from the Dow Jones Sustainability Index, as this index is based on these three pillars to categorize its metrics. After this step, the link of each of the parameter to the targets of the United Nations SDGs has been carried out. This assignment has been made based on the affinity of each of the questions, making a review one by one. The next step is to decide on the scoring method for each of the metric aspects. Numbers are fundamental and necessary for qualitative research, playing an excellent role of clarification [50]. In order to that, to obtain an index, it will be necessary to decide how to score these parameters. The purpose of this quantification is to establish levels of excellence for the companies being evaluated, aiming not only to strictly contribute to a parameter but also to improve upon it. Therefore, it is a process subject to continuous analysis, review, and improvement. Once the different parameters have been established, it will be necessary to evaluate whether any type of weighting or transformation should be made to the indices obtained, to be able to compare and evaluate the level of contribution with the United Nations SDG system. Finally, and after the scoring and transformation step, it is possible to have a company's contribution index for the different SDGs.

Proposed methodology is not airtight, so it allows considering and easily incorporating new parameters or updating those that require it, allowing a continuous improvement of the methodology, based on the requirements of interested parties.

### 2.1 Sources for the selection of metric parameters

#### 2.1.1 Mapping Mining to the SDGs: An Atlas

The atlas was developed through desk research and interviews conducted between June and August 2015 with more than 60 international experts from various sectors, including industry, civil society, governments, academia, international organizations and financial institutions.

It describes the relationship between mining and the SDGs by using examples of good practice from the sector and drawing on existing sustainable development knowledge and resources that, if replicated or scaled up, could contribute significantly to the achievement of the SDGs. This report also presents a range of challenges and opportunities that illustrate the mining sector's actual or potential contributions to achieving the SDGs, from exploration to production or, ultimately, mine closure. The primary audiences for the atlas are mining companies, their staff, management, and boards of directors. The atlas is particularly relevant for existing mines, whose operations can be adapted in terms of content to contribute to the achievement of the SDGs. It also aims to promote dialogue on how mining companies can contribute to the achievement of the SDGs, working both individually and in collaboration with governments, communities, civil society, and identified interested parties. This report selects those targets they consider to be mining-related and discards the rest, subsequently establishing indicators for each SDG and contains a chapter dedicated to each of the SDGs, focusing on the mining industry's potential contribution to meeting the goal in question, and identifying opportunities for mining companies to collaborate with other stakeholders and leverage resources to address the SDGs. This report is a useful tool to understand and promote the positive contribution of mining to the SDGs, but it does not provide concrete parameters that can be scored to contribute to the index. It also does not consider all of the SDG targets.

#### 2.1.2 Responsible Mining Index

This index provides a comprehensive reference of the main aspects of responsible mining, based on society's expectations of large-scale mining companies. It is, therefore, a clear process of consultation, participation and interaction with interested parties who experience and contribute to the impacts of business activity. The framework includes information on a set of topics, with brief descriptions of each topic as well as indicators and variables used in the RMI assessment to measure the policies and practices of mining companies in relation to these topics [47]. The assessment is based on publicly available information on companies and their mines.

**Fig. 2:**
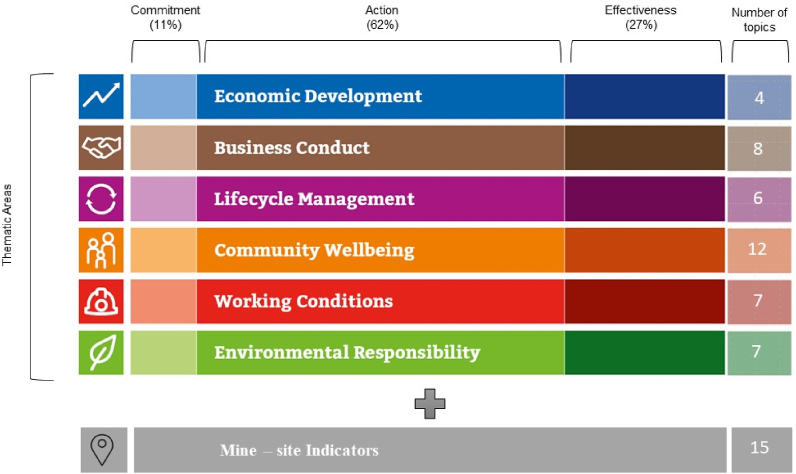
Thematic and measurement areas with weight assigned, and number of topics for RMI, including Mine-site Indicators and their number of topics. Modified from Responsible Mining Foundation, 2022.

The methodology and scope of the RMI have been developed in consultation with the Foundation's extensive network of experts and a wide range of interested parties. The RMI assessment covers 44 topics, grouped into six major thematic areas, plus an additional area for indicators specific to the mining site, and uses three types of indicators (or “measurement areas”). For each of the three types of areas, it assigns a weight to each of the three types of indicators, but this scoring and weighting system does not include the Mine-Site Indicators (Fig. 2).

The strength of this index is that it is developed especially for companies in the mining and extractive sector, so most of its indicators are directly aimed at aspects this industry.

#### 2.1.3 Dow Jones index and Corporate Sustainability Assessment

DJSI is an index that evaluates and ranks companies based on their performance in areas of environmental, social, and corporate governance [48]. DJSI is created and maintained by two companies, S&P Global and Robeco SAM. S&P Global is a leading financial services company operating worldwide. Its expertise in financial and risk analysis is combined with Robeco SAM's expertise in sustainability to assess companies in terms of their sustainable and corporate responsibility practices. The DJSI is widely recognized and used by investors and other stakeholders as an important benchmark for identifying companies with a strong focus on sustainability and corporate responsibility.

As for the questionnaire developed, the Corporate Sustainability Assessment (CSA) is a questionnaire designed to evaluate the performance of companies in terms of sustainability and corporate responsibility. This assessment is part of S&P Global’ s efforts to measure and rate the sustainability and ESG (environmental, social and corporate governance) factors of companies, which helps investors and other stakeholders to make more informed decisions [51]. The Corporate Sustainability Assessment (CSA) uses a scoring system to evaluate and rank companies based on their performance in areas related to sustainability and corporate responsibility. The CSA has a total of 3 themes (economic, social and environmental) with a total of 46 questions, 26 questions and 22 questions respectively.

### 2.2 Description of the database

**Fig. 3:**
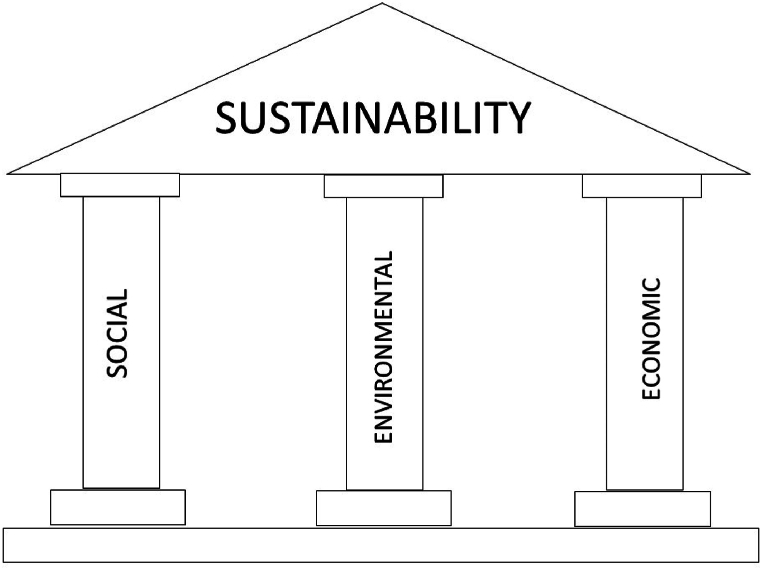
Three main pillars of sustainability (Modified form [11]).

At this point, a collection of parameters obtained from the analysis of the different data sources described is available. It will now be necessary to build the database with the parameters, the SDGs and the classic pillars of sustainability. The process of building this database began by first using the 17 SDGs of the United Nations, followed by their 169 targets. The next step was to assign each of the SDGs to one of the classic sustainability thematic, so that each of them would pertain to one of the three pillars of sustainability, the economic pillar, the social pillar, or the environmental pillar (Fig. 3).

The same SDG cannot belong to two themes at the same time. For this work, the classification used by Ref. [49] (Table 1), according to the Stockholm Resilience Centre, has been used.

**Table 1:** Classification of the SDGs according to the three pillars of sustainability. Modified from Ref. [49].

Thematic	SDG
Economic	SDG 8, SDG 9, SDG 10, SDG 12
Social	SDG 1, SDG 2, SDG 3, SDG 4, SDG 5, SDG 7, SDG 11, SDG 16, SDG 17
Environmental	SDG 6, SDG 13, SDG 14, SDG 15

The next step is to analyze the questions posed in the RMI. The RMI has a total of 94 blocks of questions to be considered for the evaluation. An interesting point of the RMI is that it associates, for each of the SDGs, the questions posed in the RMI, being able in this case to repeat the question for several SDGs. For example, question A.01.1 ″The company commits to take into account national and supranational socio-economic development plans when making business and investment decisions related to mining in producing countries, with the aim of improving socio-economic development” is associated with SDG1 End poverty, SDG8 Decent work and economic growth, SDG9 Industry, innovation, and infrastructure and SDG10 Reducing inequalities. This is interesting, as it represents the interdependence between the SDGs. The RMI associates each of its issues to each of the United Nations SDGs [47]. Table 2 shows the number of RMI questions assigned to each SDG.

**Table 2:** Number of RMI questions assigned for each of the SDGs according to Ref. [47].

SDG	Number of RMI question
1	15
2	6
3	13
4	2
5	9
6	3
7	1
8	16
9	4
10	16
11	8
12	16
13	7
14	3
15	6
16	19
17	4

There are SDGs with a higher number of questions compared to others. SDG 16 has the most questions, followed by SDG 8, while SDG 7 has only one associated question, followed by SDG 4.

Once this distribution of questions in the SDGs has been established, the next task focuses on placing each of the questions in the different targets. For this purpose, an exhaustive review and assignment has been carried out according to the expert criteria of the authors of this work.

The next step is the analysis and inclusion of the DJSI questions. In this case, as previously mentioned, the questions posed are categorized into the three main pillars of sustainability. This was the starting point for assigning the questions to the SDGs. As the SDGs were initially associated with each of these pillars, the next step was to review each of these questions and associate them with one of the SDGs within the pillar to which they belong; in this case, each question will be assigned to an SDG and there will be no repetitions. Therefore, these questions are added to the system, making up the total database. Finally, the database is composed of a series of columns within an Excel sheet that will store the following information:

SDG: Sustainable Development Goal to which the question belongs.

Target: target to which the question belongs.

Description: description of the target within each SDG.

Thematic: economic, social, or environmental.

Area: section accumulating aspects according to common topic as Economic Development, Business Conduct, Lifecycle Management, Community Wellbeing, Working Conditions, Environmental Responsibility, Mine-site Indicators.

Aspect: each of the questions asked to the target company.

Parameter: refers to the measurement indexes that each parameter must evaluate its contribution.

Aspect code: coding of each aspect to facilitate its use.

Reference: belonging of the question to the RMI system or to the DJSI system through the questions asked in the CSA.

### 2.3 Parameter scoring system and normalization function

After the database has been constructed with the information to be measured, it is necessary to establish the scoring system for each of the parameters. The scoring task is complex and is based on the evaluation of compliance with the parameters in an industry or activity. As the number of points on the scale increases, the complexity of the scoring task also increases ([52,53]). To make the score assignment as simplified as possible, without losing information, it has been decided to use a 3-level scale [54] based on Likert scales [55]. In this scoring system there is a level of non-contribution, a level of strict contribution and a level of contribution and improvement. The numerical values assigned can be seen in Table 3.

**Table 3:** Proposed scoring levels for the development of the indices and their importance in terms of contribution.

Score	Description
0	Does not contribute with this parameter
1	Contribute with this parameter
2	Contribute and improve the parameter

Once the scoring method has been established, it is necessary to see how the system responds considering the distribution of target, areas, aspects and parameters in the SDGs. The distribution of parameters is not balanced for all SDGs so, it will be necessary to adjust the numerical values for each SDG to make them comparable. Using as an example a company that has the maximum score value for all parameters, it is possible to see the maximum value for each SDG in terms of the level of contribution. In the case of the minimum, it would be 0. These data can be seen in Table 4.

**Table 4:** Score for each SDG assuming that all metric aspects are at their maximum value. The maximum score is assigned according to the scoring system proposed in this work.

SDG	Index Value
1	42
2	22
3	44
4	6
5	38
6	12
7	2
8	76
9	10
10	36
11	18
12	62
13	62
14	30
15	26
16	72
17	8

**Fig. 4:**
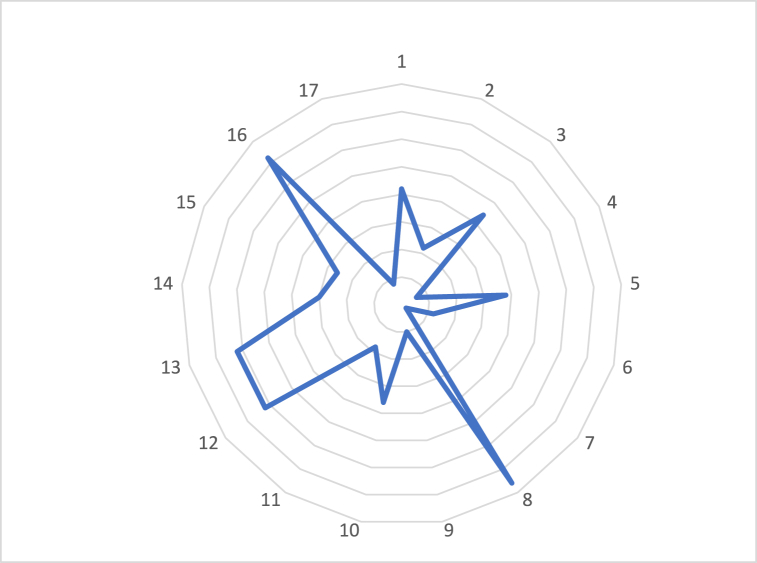
Graphical representation of the maximum index value for each SDG. In this graph it is easy to see that although all the SDGs are at their highest score (maximum contribution), each one is on a different scale of comparison.

If this were analyzed directly, the error could arise that a company complies better with some SDGs than others (Fig. 4). This effect would be derived from the weight that each SDG has in the system.

**Fig. 5:**
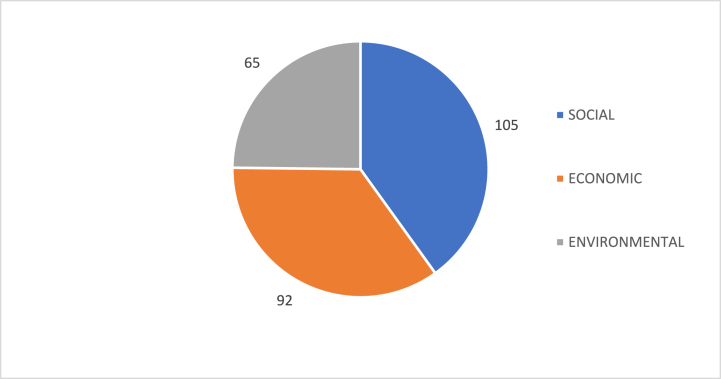
Graphical representation of the maximum index value for each theme. This image shows that each of the issues (social, economic and environmental) have different scales of measurement, as is the case with the SDGs.

If an analysis is made by theme, the same thing would happen; this result would not be comparable between the different themes since it depends directly on the weight of each one of them in the system (Fig. 5).

**Fig. 6:**
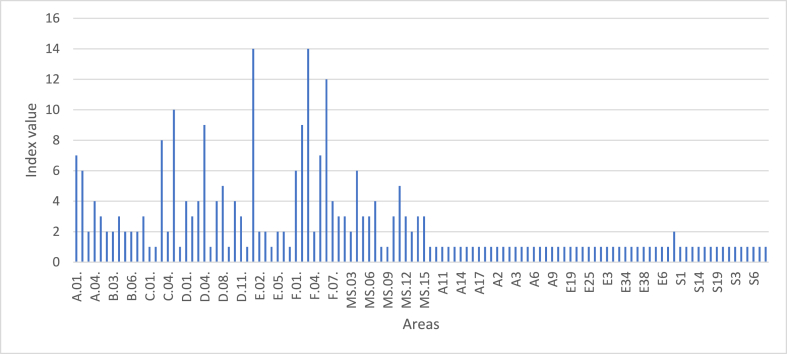
Graphical representation of the maximum index value for each area. The value of each index represents the accumulation of the indices assigned to each parameter within that area.

And if the areas derived from the aspects are analyzed, the same issue would arise: the result would not be comparable since the measurement scales are not the same (Fig. 6).

For this reason, it is necessary to transform the data to make the results comparable between SDOs and thematic areas. The question of weighting individual SDGs is difficult because assigning weights to indicators resembles a value judgment [56]. Various methods of weighting and transformation of information exist in the literature ([57,58]). In this work, this is done by applying a linear transformation (process of normalization) expression that will be applied to SDGs, thematic and areas, so that all elements contribute equally, allowing the comparison of results. To this end, a rescaling is proposed to have a common measurement system [42,44,59]. A process of linear transformations (Normalization Min-Max Scaling) [60] are now applied to the SDGs, themes and areas, considering their maximum and minimum, and the new scale chosen. Min-Max Scaling is a data preprocessing technique used to fit the values of a data set to a specific range [61]. This approach is useful in data analysis because it ensures that all attributes contribute equally to the calculations, especially in those methods sensitive to the scales of the data and facilitates the comparison of attributes with different units or scales. This normalization process implies that all data will range from 0 to 10. The general expression that has been applied to carry out this change of scale is as follows (Equation (1)):

(1) $$Ii=\frac{10*Iold}{Imax}$$

Where:

I_i_ the new transformed index.

I_old_ the index measured according to the parameters and aspects.

I_max_ the maximum index that the value to be compared can have.

**Fig. 7:**
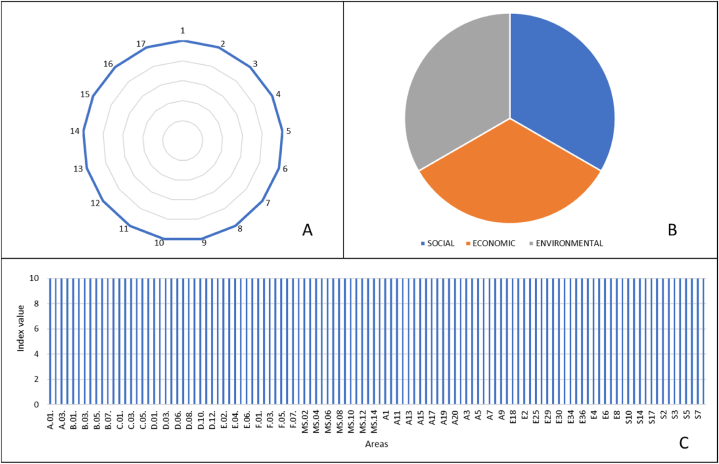
Graphical representation of the transformed indices for: A) each SDG; B) each thematic area; C) each area, assuming the maximum score for each index.

If this expression is applied to the previous example, the interpretation of the data would change. With the application of the transformation function, all the SDOs, themes and areas would be at their maximum value, which would be 10 (Fig. 7).

## 3 Results and discussion

As a result of this work, an SDG contribution assessment method for the extractive industry has been developed. This methodology proposes to fill the existing niche in the field of SDG contribution for this industry. It is a simple to use methodology for interested parties, in which, with a simple three-level scoring system, a contribution score can be formed from an SDG point of view, but also from perspective of the pillars of sustainability (three classic thematic areas). At the same time the proposed methodology provides an overview of a company's status with respect to UN policies on sustainable development. It is possible to detect in which SDGs it is necessary to make a special effort to improve, and after an analysis of its targets, aspects, and associated parameters, to implement measures that lead the company to improve its procedures in terms of sustainability. The database is made up of the 17 SDGs of the United Nations, with their 169 targets, and the different parameters and metric evaluation indices. Each SDG has been categorized within one of the three main pillars of sustainability according to the work [49], which allows a first analysis that will show how many SDGs are within each of these three main pillars. Considering these pillars of sustainability, according to the classification of [49], the 17 SDGs are distributed as follows (Table 5).

**Table 5:** Number of SDG per sustainability thematic according to the classification proposed in Ref. [49].

Thematic	Number of SDG
Economic	4
Social	9
Environmental	4

**Fig. 8:**
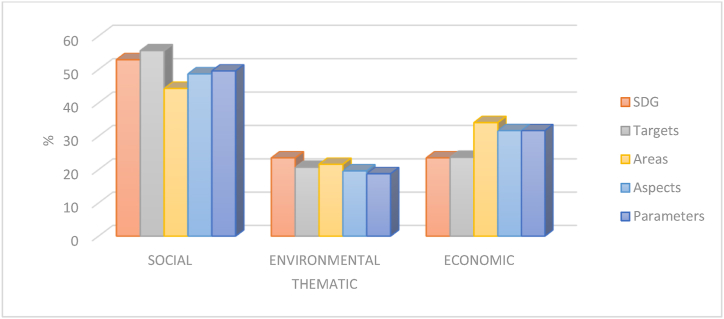
Percentage of SDGs, targets, areas, aspects and parameters per thematic. For each theme, the number of SDGs, targets, areas, aspects and parameters assigned have been evaluated and has been represented as a percentage. Own elaboration.

This means that, of the total number of SDGs, 53 percent correspond to social issues, 24 percent to environmental issues and 23 percent to economic issues. If targets are analyzed, in this case, 56 percent of the targets are social, 21 percent of the targets are environmental, and 24 percent of the targets are economic. Considering this information, and based on the thematic classification made above, the SDG system itself is balanced towards social issues. Regarding the aspects and parameters incorporated into the proposed evaluation system, it should be noted that of the total number of aspects, those coming from the RMI represent 93 percent of the total, and those coming from the DJSI represent 7 percent. This implies that the behavior of the RMI in terms of themes will be represented in the database. Starting with the parameters, there are a total of 884 parameters (metric questions) in the system, the scoring of which will result in a numerical evaluation of the 284 aspects to which they belong. If the parameters are analyzed, 50 percent of them belong to the social theme, 19 percent to the environmental theme, and 32 percent to the economic theme. In terms of aspects, social issues represent 49 percent, environmental issues represent 20 percent and economic issues represent 32 percent. Finally, evaluating the areas, there is the same behavior, with 44 percent for social issues, 22 percent for environmental issues and 34 percent for economic issues. All these data are represented in the bar chart in Fig. 8.

**Fig. 9:**
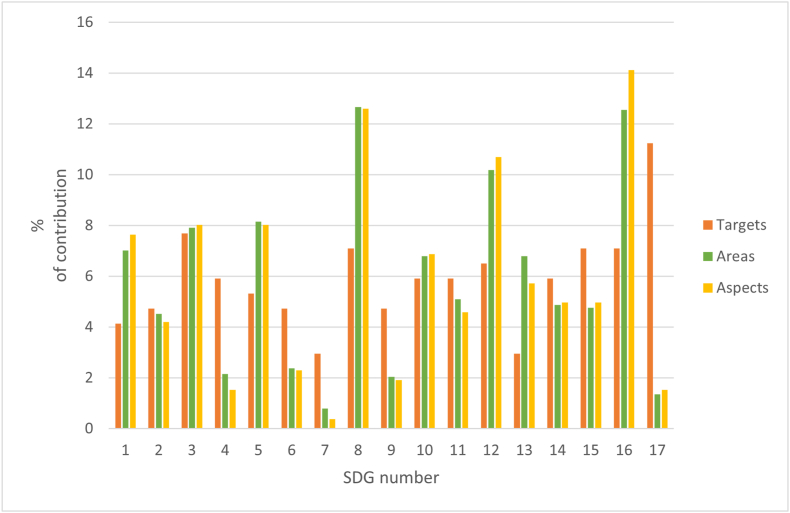
Percentage contribution of targets, areas and aspects for each of the SDGs. Percentages are calculated based on the total number of items assigned to each SDG. The X-axis shows the 17 numbered SDGs, and the Y-axis indicates the percentage contribution.

The above analysis refers to the three classic themes on which the concept of sustainability has been based. To analyze how the database behaves based on the SDGs and their interconnection [62,63], a similar analysis will be performed, considering the goals, areas and aspects. Fig. 9 compares the percentage contribution of targets, areas and aspects assigned to each of the 17 SDGs. As can be seen, SDG 17: Partnerships for the Goals and SDG 3: Good Health and Well-being, stand out above the rest, accumulating between the two of them almost 20 percent of the targets.

In terms of areas, SDG 8, SDG 12 and SDG 16 ″Promote just, peaceful and inclusive societies” once again stand out, accounting for 37 percent of the areas in the database. The same behavior of the areas is reproduced in the aspects, highlighting the same SDGs.

The distribution is heterogeneous, indicating that the system tends to prioritize certain SDGs over others. This unequal in contribution suggests that, rather than a balanced approach that treats all Sustainable Development Goals (SDGs) equally, some SDGs receive considerably more attention than others.

The fact that there is a large volume of aspects to be evaluated means that the process of analyzing the information provided requires a great deal of time, so the process will not be immediate. This makes sense since in the extractive industry there are many factors that interact directly with sustainable development, thus, the time required to perform the evaluation can be relatively long.

Regarding the scoring method, it is simple, evaluating three levels of compliance, depending on the information provided by the company to be evaluated, facilitating the task of auditing the information.

After the scoring process, it has been necessary to carry out a linear transformation of the index values by applying a Normalization Min-Max Scaling technique. This process allows direct and undistorted comparison of the results obtained between the different indices to assess, for example, the level of contribution between SDGs, between themes, or between areas. This transformation process implies that no weights have been assigned to the indices according to expert criteria, thus minimizing the subjectivity of the system. Accordingly, it does not matter if the number of parameters, aspects, areas and targets is different among the different SDGs, as at the end of the evaluation process, the measurement scales will be normalized through linear transformation, allowing for directly comparable data, avoiding subjective weights, and maintaining the interdependence of the SDG system established by the United Nations.

The analysis of the information, the scoring system and the graphical results have been carried out in Microsoft Excel. The use of this software allows the implementation of the necessary information and calculations, and at the same time it is easy to update, as required by the improvements in the sustainable development guidelines and changes in the priorities of interested parties and the context of the organization.

**Fig. 10:**
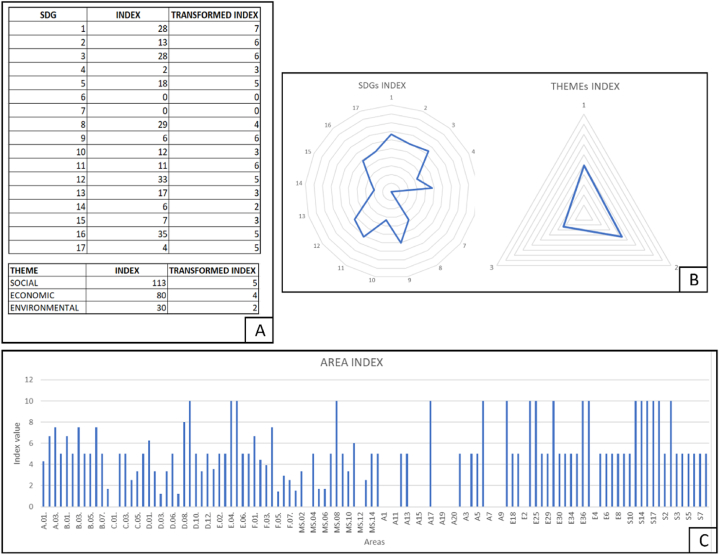
Summary image of the results obtained for a fictitious company applying the proposed methodology, including the original index and the resulting transformed index after normalization. The measurement scale for the transformed index ranges from 0 to 10. (A) The table corresponding to the original and transformed index for each SDG and also for each thematic area can be seen. (B) The graphical representation of the transformed indexes for the SDGs and thematic areas can be seen. (C) Graphical representation of the different areas according to the value of their transformed index.

To exemplify the methodology developed in this study, a fictitious company is taken as an example, which has received the index scores for each of the SDGs, as well as for the topics and areas. The information on the results for this company can be seen in Fig. 10.

In this case, the company has a low contribution (below 5 points out of 10) in SDGs 4, 6, 7, 8, 8, 10, 13, 14 and 15. Likewise, very little contribution is observed in those issues related to environmental areas (2 points out of 10), with a moderate contribution in economic and social areas (5 points out of 10 and 4 points out of 10, respectively). With these results, it is possible to address those SDGs where the company has these lower scores, analyze the responses to the evaluation aspects, and propose improvement measures that will result in a greater contribution from that company.

Regarding the scope of application of the proposed methodology, it is designed to adapt to companies of various sizes and operating contexts in the mining industry, offering flexibility and tools for its effective implementation. Its scalability is based on indicators that allow companies to address sustainability gradually. In addition, its adaptability considers the various operational contexts, and its application could offer resources to support companies in its implementation. Smaller companies can use this methodology to identify critical areas of sustainability, collaborate with partners and leverage available tools to gradually improve their performance.

## 4 Conclusions

The assessment of sustainability in European Union companies is rapidly evolving due to the region's strong commitment to climate action and sustainability. The European Union has been at the forefront of promoting corporate sustainability through increasingly stringent regulations. These regulations are expected to significantly influence how organizations evaluate and report their sustainability performance. Furthermore, there is a growing likelihood that new ISO (International Organization for Standardization) standards specific to corporate sustainability will be developed. Such standards will likely be more detailed and specific to different sectors and aspects of sustainability, which could help standardize the measurement and assessment of sustainable business performance across the EU.

Using an interdisciplinary approach, the study designed an evaluative framework that encompasses multiple dimensions, including environmental impact, social, economic, and governance aspects. The methodology was informed by metric parameters from existing studies, developed in collaboration with interested parties to incorporate expert opinions and contextual nuances of the companies' operational environments. The metrics were scored based on their level of compliance and improvement, resulting in an index reflecting the organization's contribution to the SDGs. This index was rescaled using the Normalization Min-Max Scaling method to produce a uniform scale of values for all the SDGs, enabling comparability between different indices. The simplicity of the developed methodology allows for the easy incorporation of additional parameters, simply by assigning them to a target and including them in the calculation process, facilitating continuous improvement.

The methodology provides a detailed overview of the performance of extractive companies concerning the SDGs. It identifies areas of strength as well as significant challenges these companies face on their path to sustainability. This evaluation framework offers a solid basis for implementing strategies aimed at improving their contribution to achieving the SDGs and promoting more responsible and sustainable practices. The proposed susceptibility index simplifies alignment with the SDGs by providing a clear scale from 0 to 10, allowing organization management to assess their contributions accurately. This enables them to make informed improvements to technical and business processes, take corrective actions, and anticipate potential changes in SDG compliance.

The European sustainable development policy landscape, with its increasing emphasis on promoting responsible business practices and integrating the SDGs into corporate strategies, has been closely analyzed. The alignment between European Union guidelines and the assessment proposed in this study underscores the importance of a globally consistent and harmonized approach to achieving sustainability goals. The methodology developed in this study provides a valuable tool for companies to enhance transparency, accountability, and continuous improvement in their sustainability practices. It encourages organizations to improve disclosure and take responsibility for their sustainability actions, fostering a culture of sustainability at all organizational levels.

The alignment of the proposed methodology with existing sustainability frameworks (GRI or ICMM for example) implies a strategic integration of standards and principles to promote sustainability in the mining industry. The proposed methodology promotes a higher standard of transparency and accountability in the mining industry, thereby encouraging more ethical and sustainable practices throughout the mining supply chain. This commitment to alignment with other recognized frameworks reflects a commitment to continue to improve and adapt to the evolving needs of stakeholders and the global sustainability landscape.

While this study represents a significant advance, it is important to recognize its limitations. The methodology has not yet been tested with data from real companies; instead, it has been validated using a fictitious organization with assigned parameter values to simulate its behavior concerning SDG contributions. Additionally, the thorough data collection required for this methodology may pose challenges for companies, particularly in terms of gathering all necessary information. Transparency issues can also negatively affect the assessment, and the implementation of responsible practices may involve additional costs that need to be balanced over time.

The next steps for this research include testing the methodology with data from real companies to refine the approach. Future research should focus on adapting the methodology to changes in the business environment, new sustainability demands, and potential regulations. It will also be important to develop a tool to facilitate the use of this methodology in future audits for interested companies. Long-term use of the methodology is anticipated to be a key catalyst for promoting responsible mining and extractive practices. At the corporate level, it is expected to foster a cultural change, raising awareness and commitment to sustainability across all organizational levels. By requiring transparency and accountability, this initiative encourages companies to improve their sustainability disclosure and take responsibility for their actions. Regular application of the methodology could provide a valuable feedback loop, stimulating continuous improvement and the adoption of best practices in the industry.

## CRediT authorship contribution statement

**Marta Fernández-Hernández:** Writing – review & editing, Writing – original draft, Validation, Methodology, Investigation, Conceptualization. **Pedro Mora:** Writing – review & editing, Validation, Methodology, Conceptualization. **Marcelo F. Ortega:** Writing – review & editing, Methodology, Conceptualization. **Juan Pous Cabello:** Writing – review & editing.

## Data availability statement

The data used in this research will be provided on request.

## Declaration of competing interest

The authors declare that they have no known competing financial interests or personal relationships that could have appeared to influence the work reported in this paper.
